# Effectiveness of different rotary file systems in removing the root canal filling material: A micro-computed tomography study

**DOI:** 10.34172/joddd.2021.045

**Published:** 2021-12-05

**Authors:** Esin Özlek, Hüseyin Gündüz

**Affiliations:** Department of Endodontics, Faculty of Dentistry, Van Yuzuncu Yıl University, Van, Turkey

**Keywords:** Endodontics, Micro-computed tomography, Retreatment, Root canal filling materials, Root canal preparation

## Abstract

**Background.** This study aimed to examine the retreatment efficiency of four NiTi rotary file systems with micro-computed tomography (micro-CT).

**Methods.** Forty premolar teeth were shaped up to F2 using the ProTaper Universal rotary file system and obturated with MTA Fillapex. The root canals were scanned with micro-CT to evaluate the volume of root canal filling before retreatment. The samples were randomly divided into four groups according to the file system used to remove root canal filling material (n=10): ProTaper Universal Retreatment, ProTaper NEXT, EdgeFile XR, and EdgeFile® X3 NiTi system. All the samples were scanned with a micro-CT device for the second time to evaluate the amount of residual filling material in the root canals. The percentages of filling material removed from root canals were calculated. Additionally, the time spent on the removal of the root canal filling material was recorded. The data were analyzed using the Shapiro-Wilk and Kruskal-Wallis tests.

**Results.** There were no significant differences between the groups in the percentage of root canal filling material removal. However, a statistically significant difference was found between the groups in the time required to reach the apex and remove the entire filling material. The time required to remove the root canal filling material was higher in the EdgeFile® X3 group.

**Conclusion.** NiTi files manufactured for root canal preparation can be used effectively and safely to remove root canal filling materials. EdgeFile XR produced for retreatment can be used as an alternative to ProTaper Universal Retreatment files.

## Introduction

Endodontic therapy aims to create healthy periapical tissues and maintain their health. For this, it is necessary to remove the infected tissue in the root canal cavity as much as possible, disinfect the root canals, and block them as effectively as possible.^1^ In recent years, many studies have evaluated the success of endodontic treatment. As a result of these studies, success rates varying between 68% and 98% have been reported.^2,3^ Despite high success rates in first-time root canal treatments, unsuccessful endodontic treatments are also reported.^4^

Non-surgical root canal retreatment, apical surgery, and intentional replantation options are available as solutions for unsuccessful root canal treatments. Non-surgical root canal retreatment is the most preferred option among these treatments because it is less invasive and has a higher success rate.^5^ Removal of root canal filling materials from root canals is an essential step in the success of retreatment because the filling materials remaining in the root canal walls cause microorganisms to settle and multiply. In addition, remaining filling materials decrease the efficacy of irrigation solutions and prevent the penetration of the new filling material, negatively affecting the success of the treatment.^6^

In recent years, the use of NiTi rotary files for removing root canal filling has gained popularity thanks to its advantages, such as shortening the treatment process, easing the application, and being more flexible than hand files.^7^ As an alternative to NiTi retreatment file systems used to remove root canal filling from canal walls, NiTi files are also recommended to shape root canals.^8,9^

ProTaper Universal Retreatment (PTUR; Dentsply, Maillefer, Ballaigues, Switzerland) files are characterized by a progressive taper, a convex triangular cross-section, and a modified guide tip. It consists of three files (D1, D2, and D3) with various tapers and diameters at the tip. The D1 file has an active tip structure that facilitates the penetration of subsequent files. The inactive ends of D2 and D3 reduce the possibility of stepping, perforation, and stripping during the removal of the root canal filling material.^10^

ProTaper Next (PTN; Dentsply, Maillefer, Ballaigues, Switzerland) is a NiTi file system with a rectangular cross-section design, produced from M-Wire alloy, and works with a continuous asymmetric rotation movement. In addition, the offset design facilitates the removal of debris and filler from the canal and increases the flexibility of PTN files along the active part.^11^

EdgeFile XR file system is made of NiTi alloy named Fire-Wire^TM^. This alloy improves the deformation and strength features of the metal, increasing the flexibility, performance, and durability of the file. This improves and accelerates endodontic retreatment. The system consists of four files, including R1 (25.12), R2 (25.08), R3 (25.06), and R4 (25.04), which are used in the crown-down technique. All the files have fixed tapers and parabolic sections.^12^ The EdgeFile X3 system features tools manufactured by a heat treatment process (FireWire NiTi) that increases the flexural strength and flexibility of NiTi tools customized for the manufacturer.^7^ EdgeFile X3 files have fixed.06 tapers, parabolic cross-sections, and variable helix angles.^13^

Previous studies have reported that file systems produced for retreatment cannot completely remove the root canal filling materials.^14,15^ However, there are limited studies in the literature showing the retreatment efficiency of file systems produced for root canal preparation.^8,9^ Therefore, in this study, file systems with different metal properties (M-Wire, FireWire) and different areas of use (retreatment, root canal preparation), i.e., PTUR (Dentsply, Maillefer, Ballagues, Switzerland), EdgeFile XR NiTi (EdgeEndo, USA), PTN (Dentsply Maillefer, Baillagues, Switzerland), and EdgeFile® X3 (EdgeEndo, USA), and their root canal filling removal efficiency were evaluated. Thus, file systems used in root canal preparation aim to remove the root canal filling and complete the root canal preparation with a single file system without needing another file system. In addition, during root canal retreatment, the time taken to reach the apex and remove the entire filling was calculated by its measurement with a stopwatch. The null hypothesis of this study is that the use of different rotary file systems during root canal retreatments does not result in a significant difference in the efficiency of the root canal filling material removal and treatment duration.

## Methods

### Selection of samples

In this study, 40 single-rooted mandibular premolar teeth extracted for periodontal or orthodontic reasons were used. The number of samples in the study was calculated with reference to the studies in the literature.^6,16^ Teeth without caries, with a single root, a single canal, and complete root development were included in this study. The mesiodistal and buccolingual radiographs were taken, and the root canal anatomy of the teeth was examined. Teeth with a single root, closed apex, and similar root canal widths were included in the study to provide standardization.

After stereomicroscopic examination, teeth with cracks or fractures, >10º curvatures in the root canal, calcification, and resorption were excluded from the study. The teeth were stored in distilled water at room temperature until they were used. The tooth crowns were removed with a diamond saw under water cooling, ensuring a root length of 15 mm. Access cavities were prepared with a diamond round bur (Bosphorus, Istanbul, Turkey). A #10 K-file (Mani Inc., Tochigi, Japan) was placed in the root canal, and the working length was determined 1 mm shorter than the length seen at the apical foramen. Teeth with an apical width greater than the tip diameter of a #15 K-file (Mani Inc, Tochigi, Japan) were excluded from the study.

### Shaping and obturation of root canals

ProTaper Universal (Dentsply Maillefer, Baillagues, Switzerland) rotary file system was used in the shaping process of root canals at 300 rpm and 3 Ncm torque following the manufacturer’s instructions. The expansion was made with SX, S1, S2, F1, and F2 files, respectively, with F2 apical width, using the X-Smart endomotor (Dentsply Maillefer, Ballaigues, Switzerland). In the forming and expansion process, the samples were irrigated with 2 mL of 5.25% NaOCl (Microvem, Istanbul, Turkey) solution in each file change. In the final irrigation process, 5 mL of 17% EDTA (Imicryl, Konya, Turkey), 5 mL of 5.25% NaOCl, and 5 mL of distilled water were used, respectively. The root canals were then dried with F2 angled paper points (Dentsply Maillefer, Baillagues, Switzerland). Next, the root canals of all the samples were filled with F2 gutta-percha (Dentsply Maillefer, Baillagues, Switzerland) and MTA Fillapex (Angelus, Londrina, Brazil) using a root canal sealer with the single-cone technique. The access cavities were covered with a temporary filling material (Cavit, 3M ESPE, Germany). Then, the roots were embedded in acrylic resin (Procryla, President Dental, Munich, Germany) to obtain the images of the samples in the standard position during micro-computed tomography (micro-CT) scans.

The radiographic quality of the root canal filling was checked by periapical radiographs taken from the buccolingual and mesiodistal angles. Samples with insufficient root canal filling were excluded from the study. All the samples were stored for one month at 37ºC under 100% humidity in the drying oven (Memmert UN 110) to allow the root canal filling material to harden.^8^

### Calculation of root canal filling volume with micro-CT

The samples prepared by being embedded in acrylic resin were placed in the micro-CT scanning device. Root canal fillings were scanned to evaluate the volume using Scanco Medical Micro-CT 50 device (Scanco Medical, Brüttisellen, Switzerland) before the root canal retreatment. Scans were carried out at 25 µm isotropic resolution, 90 kVp, and 155 mA; 0.1-mm Cu filter was used in scans and realized with a rotation angle of 0.6° and a total vertical rotation angle of 180°. The scan of a sample took 28 minutes on average. At the end of the scans, 640 raw images were obtained for each sample and saved in the TIFF format. Image reconstruction was performed using CT Evaluation Program (V6.5, Scanco Medical, Switzerland), and cross-sectional images were obtained. Initial root canal filling volumes (mm^3^) were calculated.

### Removal of root canal filling material

After scanning the root canals with micro-CT to calculate the root canal filling volumes, the samples were randomly divided into four groups according to the file system to be used to remove the root canal filling (n = 10).

**Group 1:** ProTaper Universal Retreatment NiTi System (n = 10)

The root canal filling of the samples in this group was removed with the ProTaper Universal Retreatment NiTi rotary file system. All the files were used at 3-Ncm torque and 500-rpm speed according to the manufacturer’s recommendations. D1 (30/.09), D2 (25/.08), and D3 (20/.07) files were used to remove the root canal filling in the coronal, middle, and apical thirds, respectively, until the file reached the working length. The working time was recorded after the D3 file reached the working length (T1). Later, until no gutta-percha or sealer came out of the root canal, the D2 (25/.08) file was used in the working length, and the root canal was cleaned; then, the total time was recorded (T2).

**Group 2:** ProTaper NEXT NiTi System (n = 10)

Root canal filling of the samples in this group was removed by the ProTaper NEXT NiTi rotary file system. In the coronal and middle thirds of root canals, X3 (30/.07) file was used; in the apical third, X2 (25/.06) file was used until it reached the working length.^14^ The files were used at 500-rpm speed and 3-Ncm torque. The working time was saved when X2 (25/.06) file reached the working length (T1). Later, X2 (25/.06) file was used at the working length until no gutta-percha or sealer came out of the root canal, and the root canal was cleaned; then, the total time was recorded (T2).

**Group 3:** EdgeFile XR NiTi System (n = 10)

Root canal filling of the samples in this group was removed by the EdgeFile XR with NiTi files. R1 (25/.12), R2 (25/.08), R3 (25/.06), and R4 (25/.04) files were used with the crown-down method at 500-rpm speed and 3-Ncm torque according to manufacturer instructions, by moving them 2-4 mm in the root canal filling at a light and medium pressure, respectively. This process was repeated until the R4 (25/.04) file reached the working length, and the working time was recorded (T1). Finally, R3 (25/.06) file was used at the working length until no gutta-percha or sealer came out of the root canal, and the root canal was cleaned; then, the total time was recorded (T2).

**Group 4:** EdgeFile® X3 NiTi System (n = 10)

Root canal filling of the samples in this group was removed by the EdgeFile XR with NiTi files. C3 (30/.06) file was used in the coronal and middle thirds of root canals; in the apical third, C2 (25/.06) file was used at 500-rpm and 3-Ncm torque until they reached the working length, and the working time was recorded (T1). Finally, C2 (25/.06) file was used at the working length until no gutta-percha or sealer came out of the root canal, and the root canal was cleaned; then, the total time was recorded (T2).

In all the samples, after each file change, 2 mL of 5.25% NaOCI was used to remove the root canal filling, followed by 5 mL of 17% EDTA and 5 mL of 5.25% NaOCI were used as the final irrigation. The root canals were then irrigated with 5 mL of distilled water and dried with paper points. The access cavities were closed with the temporary filling material Cavit (3M ESPE, St Paul, MN, USA). Each file was used only in one tooth.

### Micro-CT scans of teeth after removing root canal filling material

**Figure 1:**
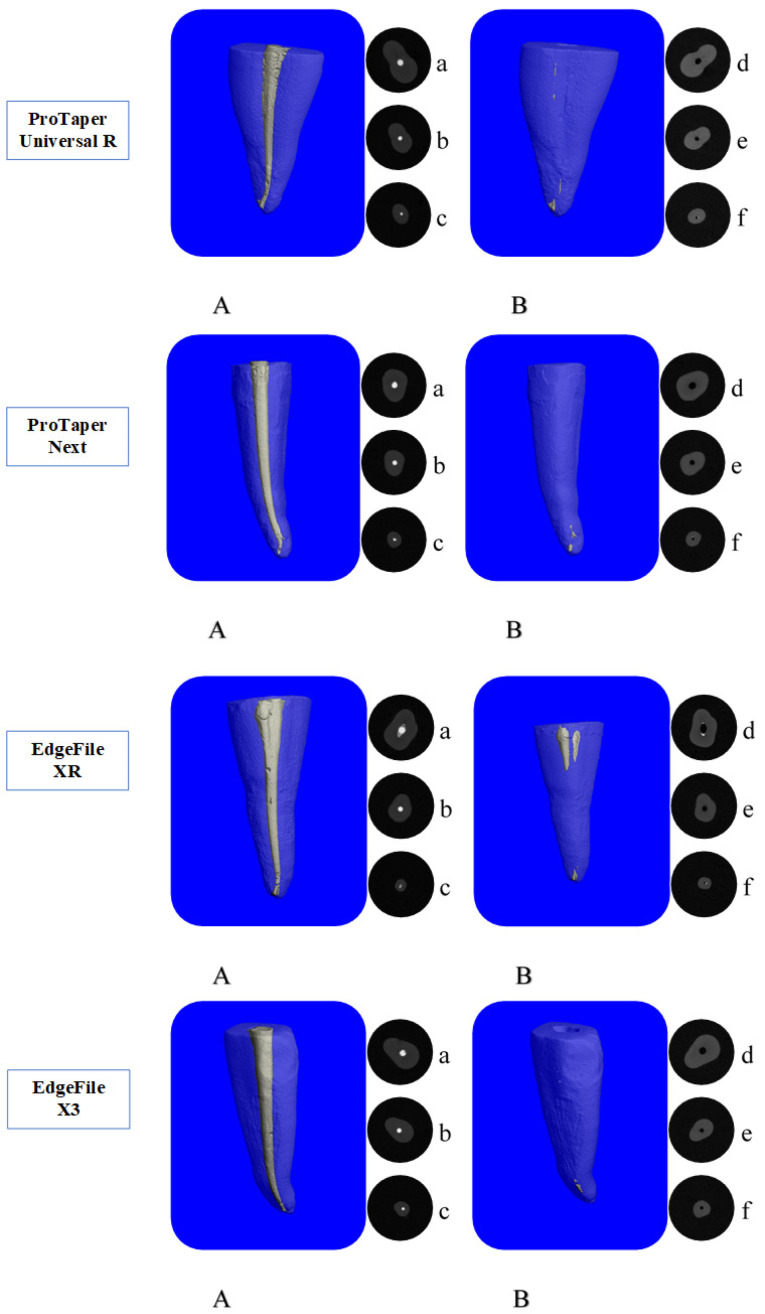
Three-dimensional reconstruction (A, B) and horizontal section images (d, e, f) of the axial section of the samples belonging to the Protaper Universal R, ProTaper NEXT EdgeFile XR, and EdgeFile X3 groups after Micro-CT scans. (A) After root canal filling and (B) after removing the root canal filling material. Coronal, middle, and apical horizontal section images after micro-CT scans: (a, b, c) after root canal filling and (d, e, f) after removing the root canal filling material.

After removing the root canal filling materials with ProTaper Universal Retreatment NiTi system, ProTaper NEXT NiTi system, EdgeFile XR NiTi system, and EdgeFile® X3 NiTi system, to examine the filling material remaining in the root canals, all the samples were scanned with the Scanco Medical Micro-CT 50 CT device using the same parameters for the second time. The remaining volume of the root canal filling material after the procedure was calculated (Figure 1). Then, the root canal filling percentages were calculated using the remaining filling material volume values before and after the procedure.

### Root canal retreatment time

Time to reach the apex (T1) was defined as the time from the first instrument used to enter the root canal until the working length was calculated by its measurement with a stopwatch. Time taken to remove all the filling (T2) was determined after reaching the working length and was defined as the time passed until the root canal filling did not come out with the last root canal file as measured with a stopwatch. These times were not included in the time spent during the instrument change and irrigation.

### Statistical analysis

The data were analyzed with IBM SPSS 23. Compatibility with normal distribution was assessed by the Shapiro-Wilk test. The Kruskal-Wallis test was used to compare the values of the percentage of removed filling that did not show a normal distribution, the time to reach the apex, and the time to remove the whole filling material. Multiple comparisons were taken into account regarding the corrected *P* value. Data were presented as medians (min-max). *P* value <0.05 was considered statistically significant.

## Results

### Findings related to the percentage of filling material removed from root canals

The median, maximum and minimum values of the percentage (%) of the filling material removed from the root canals after root canal retreatment are presented in Table 1. According to the Kruskal-Wallis test results, there was no statistically significant difference between the groups (*P* > 0.05).

**Table 1:** Percentage of root canal filling removed (%)

	**n**	**Median**	**Minimum**	**Maximum**
Protaper Universal R	10	97.69	90.92	99.45
ProTaper NEXT	10	99	93.4	99.86
EdgeFile XR	10	98.94	96.74	99.94
EdgeFile® X3	10	96.56	64.51	99.88

*P* = 0.146

### Results of the root canal retreatment time

Table 2 presents the time to reach the apex (T1) and the time to remove the entire filling material (T2) during the removal of the root canal filling material. A statistically significant difference was found between the groups in the time taken to reach the apex (*P* = 0.001). EdgeFile® X3 system median value was higher than those of ProTaper Universal Retreatment system, EdgeFile XR system, and ProTaper NEXT system. There was no statistically significant difference between the median values of the ProTaper NEXT system and the others (*P***=**0.001).

**Table 2:** T1 and T2 times by groups

	**n**	**T1**	**T2**
Protaper Universal R	10	46.9 (0 – 56.2)^b^	61.5 (0 – 71.2)^b^
ProTaper NEXT	10	55.3 (0 – 80.8)^ab^	77.2 (0 – 112.1)^ab^
EdgeFile XR	10	51.1 (41.4 ‒ 58.5)^b^	69.8 (0 – 75.3)^b^
EdgeFile® X3	10	80.6 (0 ‒ 108.8)^a^	101.3 (75.8 – 152.8)^a^
P*		0.001	<0.001

a-b: There was no difference between groups with the same letter within each time interval. *Kruskal Wallis. T1: Time taken to reach the apex (s). T2: Time taken for removal of the whole filling (s).

A statistically significant difference was found between the groups when the time taken to remove the whole filling material was considered (*P* < 0.001). EdgeFile® X3 NiTi system median value was higher than the median values of both the ProTaper Universal Retreatment NiTi system and EdgeFile XR NiTi system. There was no difference between the ProTaper NEXT NiTi system median values and the others (*P* < 0.001).

## Discussion

In root canal retreatment, it is necessary to remove all the root canal filling material to remove bacterial residues, increase the effectiveness of irrigation solutions and intracanal medicaments on root canal dentin, and ensure the sealing of the new root canal filling.^15^ This study showed that no file system was able to remove the root canal filling material completely, consistent with similar studies.^8,9,16^ Although there were no statistically significant differences between the groups, the highest amount of root canal filling material was removed in the PTN group. Thus, the null hypothesis of the study was partially rejected.

Özyürek and Demiryürek^14^ compared the root canal retreatment efficacy of PTUR, PTN, TF Adaptive systems and the Reciproc system. PTUR and PTN systems exhibited a similar effect, but the remaining root canal filling material was less in the PTUR system. It has also been reported that these two systems are more effective than other systems. However, considering this fact, Martinset al^8^ and Nevareset al^9^ also reported that the PTN system is an effective system for removing root canal filling material.The PTN system can be effective because it creates an expanded area due to its cross-section design, facilitating the removal of root filling material and debris. The smaller contact area between the instrument and the root canal walls reduces the possibility of fracture in the files and provides space for coronal extrusion of gutta-percha. In this study, although no statistically significant difference was found between the efficiency of PTUR and PTN systems in removing the root canal filling, the PTN system removed more root canal filling material than the PTUR system.

In the study by Özyürek and Demiryürek,^14^ cross-sections were photographed, and digital images were obtained as an evaluation method. In the present study, micro-CT was used as an evaluation method. We believe that the contradictory results between the two studies are due to the different methodologies used and that the three-dimensional analysis methods provide more accurate results because the residues remaining in the dentinal tubules cannot be identified in two-dimensional examinations.

Some studies have compared the efficiency of the root canal filling material removal with file systems produced for root canal retreatment and with file systems produced for root canal preparation. These studies have shown that the efficiency of root canal filling material removal by files used for root canal preparation is similar to or more effective than files produced for root canal retreatment.^16^ In this study, the PTN file system used in root canal preparation removed more root canal filling material than PTUR and EdgeFile XR file systems produced specifically for root canal retreatment. These results show that files used for root canal preparation can be used instead of file systems produced for root canal retreatment, thanks to their cross-sectional design and metal alloys.

In the present study, EdgeFile XR system removed more root canal filling material than PTUR and EdgeFile® X3 systems. This might be related to the metal alloy of the EdgeFile XR system and that this alloy increases the efficiency and physical properties of the file, as reported by the manufacturer. In the literature, there are a limited number of studies investigating the effectiveness of the EdgeFile XR system in removing the root canal filling material.^13^ Since no studies evaluating the efficacy of root canal retreatment of this system with PTUR and PTN file systems were found, no comparisons could be made. Tomeret al^13^ compared the efficacy of EdgeFile XR, Mtwo, R-Endo files in removing the root canal filling material. They reported that the EdgeFile XR file system was more effective in removing the canal filling material than other groups, and it was claimed that the better performance of EdgeFile XR files is due to improvements in the physical properties of the files as a result of the Fire-Wire^TM^ heat treatment.

In this study, the efficiency of the EdgeFile X3 file system in removing the root canal filling material did not differ significantly from other file systems. Similar studies have been conducted on the root canal filling removal efficiency of file systems produced for root canal preparation. These studies have shown that file systems produced for root canal preparation can effectively remove the root canal filling material.^17,18^ In this study, EdgeFile X3 and PTN system showed similar efficiency in removing the root canal filling. However, PTN removed more root canal filling material. This might be due to the flexibility of the EdgeFile X3 system thanks to its heat-treated metal properties and consequently the low penetration of the file into gutta-percha.

In this study, the time to reach the apex (T1) and the time to remove all the filling material (T2) during the removal of the root canal filling material in the EdgeFile X3 system were significantly longer than the other file systems. The reason for this might be the high flexibility of this file system due to Fire-Wire technology and the low penetration into the gutta-percha due to its inactive tip structure.^19^ The PTUR group had shorter T1 and T2 times than other file groups. The active tip of the D1 file in the PTUR system facilitates its penetration into the root canal filling, and thanks to negative and positive cutting angles, root canal filling can be removed quickly and effectively.^19,20^ These findings are consistent with the study carried out by Özyürek and Demiryürek^14^ and Yilmazet al.^16^ In the present study, the EdgeFile XR system had a shorter operating time than the PTN system because this file system is resistant to fracture due to its parabolic cross-section. However, it is an extremely effective and flexible tool. No comparisons could be made since no studies have evaluated the EdgeFile XR system with PTUR and PTN systems in terms of the duration of the root canal filling material removal. This study showed that the PTN system required shorter T1 and T2 time than the EdgeFile X3 group. Nevareset al^9^ showed that the PTN system is an effective method for removing the root canal filling material in terms of time. The reason for this can be explained as follows: the rectangular cross-section and asymmetrical rotation movement of the PTN system creates an area to remove the root canal filling material, shortening the duration of the procedure.

The amount of material remaining after root canal treatment has been evaluated by many methods so far. These methods include radiography,^21^ sectioning,^17^ dental operation microscopy,^22^ scanning electron microscopy,^23^ CT,^24^ cone-beam computed tomography (CBCT),^18^ and micro-CT.^6,16,19^ Micro-CT has advantages such as not causing structural changes and material loss, repeatability, and the possibility of three-dimensional structuring and evaluation.^25^ Recently, micro-CT imaging has been used frequently to evaluate the removal of the root canal filling material because this method provides a detailed evaluation of the 3D morphological features of the studied object both quantitatively and qualitatively. It also allows for the analysis of different stages of the experiment, as samples are preserved and reused.^8^ In this study, micro-CT evaluation was preferred because of its advantages and because it has the highest accuracy among current techniques.

## Conclusions

Within the limitations of this study, no file system used was able to remove the root canal filling material completely. PTN and EdgeFile X3 file systems produced for root canal preparation exhibited efficiency similar to PTUR and EdgeFile XR file systems in removing the root canal filling material. However, the PTN file system removed more root canal filling material than EdgeFile XR, PTUR, and EdgeFile X3 file systems. EdgeFile XR system, which was produced for the root canal filling material removal, had an effect similar to the PTUR system. Therefore, EdgeFile XR can be used as an alternative to the PTUR system to remove the root canal filling material.

## Authors’ Contributions

All the authors contributed to the study concept and design. Material preparation, data collection, and analysis were performed by HG. The first draft of the manuscript was written by EO, and all authors commented on previous versions of the manuscript. All the authors read and approved the final manuscript.

## Acknowledgments

None.

## Funding

This Project was supported by Van Yüzüncü Yıl University Scientific Research Coordinator, Project No: TDH-2019-7925.

## Competing Interests

The authors declare no competing interests with regards to the authorship and/or publication of this article.

## Ethics Approval

This study was approved by the Van Yüzüncü Yıl University Clinical Research Ethics Committee with the decision numbered 2018/11 and dated 21.12.2018.
